# The SARS-CoV-2 receptor, ACE-2, is expressed on many different cell types: implications for ACE-inhibitor- and angiotensin II receptor blocker-based cardiovascular therapies

**DOI:** 10.1007/s11739-020-02364-6

**Published:** 2020-05-19

**Authors:** Adriana Albini, Giovanni Di Guardo, Douglas McClain Noonan, Michele Lombardo

**Affiliations:** 1 Scientific and Technology Pole, IRCCS MultiMedica, Milan, Italy; 2 Faculty of Veterinary Medicine, University of Teramo, 64100 Teramo, Italy; 3 Department of Biotechnology and Life Sciences, University of Insubria, Varèse, Italy; 4 Cardiology Unit, San Giuseppe Hospital-MultiMedica, Milan, Italy

**Keywords:** COVID-19, Cardiovascular disease, Thrombosis, Endothelium, Angiotensin II receptor blockers, ACE-inhibitors

## Abstract

SARS-CoV-2 is characterized by a spike protein allowing viral binding to the angiotensin-converting enzyme (ACE)-2, which acts as a viral receptor and is expressed on the surface of several pulmonary and extra-pulmonary cell types, including cardiac, renal, intestinal and endothelial cells. There is evidence that also endothelial cells are infected by SARS-COV-2, with subsequent occurrence of systemic vasculitis, thromboembolism and disseminated intravascular coagulation. Those effects, together with the “cytokine storm” are involved in a worse prognosis. In clinical practice, angiotensin-converting enzyme inhibitors (ACE-Is) and angiotensin II receptor blockers (ARBs) are extensively used for the treatment of hypertension and other cardiovascular diseases. In *in vivo* studies, ACE-Is and ARBs seem to paradoxically increase ACE-2 expression, which could favour SARS-CoV-2 infection of host’s cells and tissues. By contrast, in patients treated with ACE-Is and ARBs, ACE-2 shows a downregulation at the mRNA and protein levels in kidney and cardiac tissues. Yet, it has been claimed that both ARBs and ACE-Is could result potentially useful in the clinical course of SARS-CoV-2-infected patients. As detected in China and as the Italian epidemiological situation confirms, the most prevalent comorbidities in deceased patients with COVID-19 are hypertension, diabetes and cardiovascular diseases. Older COVID-19-affected patients with cardiovascular comorbidities exhibit a more severe clinical course and a worse prognosis, with many of them being also treated with ARBs or ACE-Is. Another confounding factor is cigarette smoking, which has been reported to increase ACE-2 expression in both experimental models and humans. Sex also plays a role, with chromosome X harbouring the gene coding for ACE-2, which is one of the possible explanations of why mortality in female patients is lower. Viral entry also depends on TMPRSS2 protease activity, an androgen dependent enzyme. Despite the relevance of experimental animal studies, to comprehensively address the question of the potential hazards or benefits of ACE-Is and ARBs on the clinical course of COVID-19-affected patients treated by these anti-hypertensive drugs, we will need randomized human studies. We claim the need of adequately powered, prospective studies aimed at answering the following questions of paramount importance for cardiovascular, internal and emergency medicine: Do ACE-Is and ARBs exert similar or different effects on infection or disease course? Are such effects dangerous, neutral or even useful in older, COVID-19-affected patients? Do they act on multiple cell types? Since ACE-Is and ARBs have different molecular targets, the clinical course of SARS-CoV-2 infection could be also different in patients treated by one or the other of these two drug classes. At present, insufficient detailed data from trials have been made available.

Type 2 pneumocytes and ciliated bronchial epithelial cells, the main cells within the respiratory system are targeted by Severe Acute Respiratory Syndrome (SARS) Coronaviruses (CoVs), namely SARS-CoV and the recently identified SARS-CoV-2, allow viral entry through the angiotensin-converting enzyme 2 (ACE-2) [1–4]. ACE-2 expression is not limited to the respiratory system, being very represented also in other tissues like gut, heart, kidney as well as arterial and venous endothelial cells in all organs studied [5] (Fig. 1). Similar to human immunodeficiency virus (HIV), the receptors and co-receptors are expressed also on cells of haematopoietic origin [6], SARS-CoV-2 is able to infect and cause damage to T lymphocytes, despite their very low expression levels of ACE-2, which argues in favour of an alternate receptor allowing viral entry into these cells, where the virus is not able to replicate [7].

**Fig. 1:**
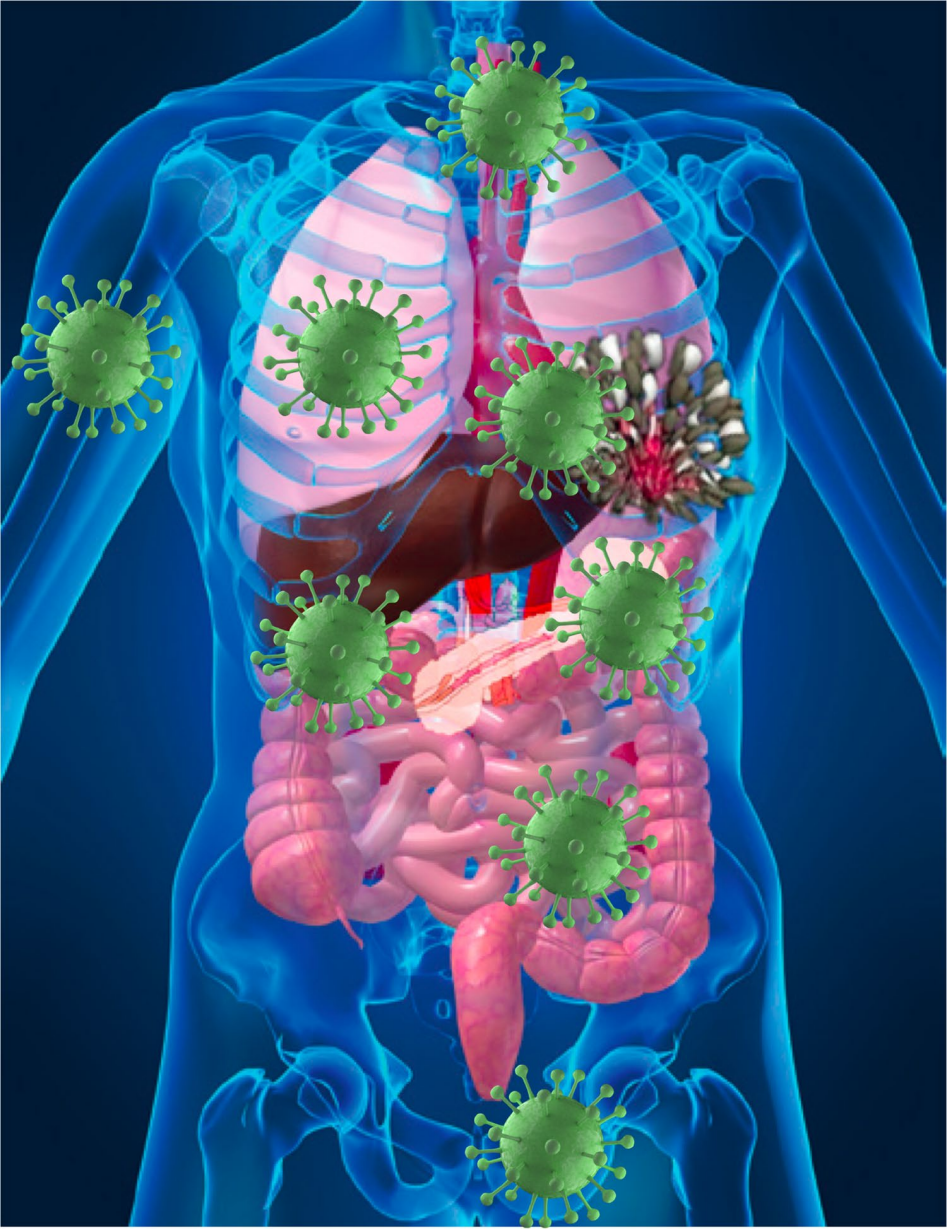
ACE-2 is expressed on lung, gut, kidney epithelial cells, cardiac, endothelial cells and in testis and to lesser extent in the breast, skin and other organs

Among SARS-CoV-2-infected patients in Italy who died until April 23 and with a median age of 80 years, 69% of them were hypertensive, 31% had type 2 diabetes, 27% suffered from ischemic heart disease, 21% from atrial fibrillation, and 16% from heart failure, with 82% of them showing 2 or more comorbidities [8]. This could contribute to explain the apparently much higher (around 13%) COVID-19-related case-fatality rates in Italy, as compared to those reported [9] in cohorts of China patients (around 5%). In China the lethal cases of SARS-CoV-2 infection have occurred in patients with a median age of 63 years [10], with their most prevalent comorbidities being represented, albeit to a lower extent, by hypertension (24%), diabetes (16%) and cardiovascular conditions (9%) [11, 12]. Among 201 SARS-CoV-2-infected patients, (average age 51 years), 42% developed severe acute respiratory distress syndrome, 27% had hypertension, and 19% were diabetic [13].

In one retrospective study on 416 COVID-19-affected patients, those with myocardial injury (transient increase of hs-T-troponin values) had significantly higher mortality than the subgroup without myocardial injury (51% vs 4.4%, respectively; *p *< 0.001) [14]. In another report on 187 COVID-19-affected patients, 23% of whom died, the mortality rate during hospitalization was, respectively, 7.6% among the patients without cardiovascular disease and no elevation of hs-T-troponin levels, 13.3% among those with an underlying cardiovascular disease but normal troponin levels, 37% for those without previous cardiovascular disease but elevated troponin levels, and 69% among those showing both cardiovascular disease and an increased hs-T-troponin profile [15].

A high frequency of thrombosis and thromboembolism has been additionally reported in COVID-19-affected patients [16–18] (Fig. 1). ACE-2 expression has been demonstrated in endothelial cells from arterial and venous vessels [5] and there is clear-cut evidence that endothelial cells are prone to acquire SARS-CoV-2 infection [19], with subsequent development of endotheliitis, endothelial cell damage, systemic vasculitis and disseminated intravascular coagulation (DIC). COVID-19-affected patients with acute respiratory failure present a severe hypercoagulability rather than consumptive coagulopathy [20], with a concurrent massive elevation of von Willebrand Factor [21] towards massive endothelial stimulation and damage with release of von Willebrand Factor from Weibel–Palade bodies. In Chinese, Italian and French cohorts of COVID-19-affected patients, a substantial increase in D-dimer and fibrin degradation products, along with a longer prothrombin time and activated partial thromboplastin time has additionally been documented as compared to uninfected patients [18, 20, 22]. Significantly higher levels of D-dimer and fibrin degradation products, along with longer prothrombin and activated thromboplastin times were observed in SARS-CoV-2-infected subjects who did not survive, with the 71.4% frequency of DIC in non-survivors and 0.6% in survivors highlighting the critical role played by such condition in COVID-19 severity and associated deaths [23].

All the above pathogenetic mechanisms, in fact, are increasingly known to make a significant contribution to the occurrence of severe disease patterns and COVID-19-associated mortality [24].

The aforementioned findings raise the following concerns:

1. COVID-19-affected patients with cardiovascular morbidity, hypertension and diabetes show a worse prognosis, particularly in advanced age.
2. Many of these patients, in Italy, are currently treated with angiotensin II receptor blockers (ARBs) or ACE-inhibitors (ACE-Is) [25].
3. SARS-CoV-2 is known to damage the cardiovascular system, thereby causing myocarditis [26], myocardial injury, coronary plaque instability, and type 1 myocardial infarction [27], along with oxygen supply and demand imbalance, systemic vasculitis, disseminated intravascular coagulation (DIC) [14, 22, 23], and heart failure [14, 28].
4. Renin–angiotensin–aldosterone system (RAAS)-interfering drugs are likely to affect ACE-2 receptor-SARS-CoV-2 interaction dynamics within lung, heart, vascular, kidney and gut tissues [5, 19], while it is still not completely elucidated how such interactions are relevant to the clinical course of cardiovascular comorbidities in patients with COVID-19 [29].

Are these two drug classes (ARBs, ACE-Is or other anti-hypertensive drugs) acting differently from each other in the pathogenetic evolution of SARS-CoV-2 infection? Do they affect infection of different cell types?

It has been demonstrated that human ACE-2 enzyme is a negative regulator of RAAS [30, 31], thus providing a crucial link between immunity, inflammation, increased coagulopathy, and cardiovascular disease, thereby serving as a protective mechanism against heart failure, myocardial infarction, lung disease, hypertension, vascular permeability, and diabetes [24, 32] (Fig. 2). ACE-2 function, following SARS-CoV binding, is reduced due to endocytosis and proteolytic cleavage [33] and there are high levels of Ang II in the blood of patients with COVID-19 [34, 35]. Consequently, the up-regulation of human ACE-2 induced by RAAS-antagonists in SARS-CoV-2-infected patients could be clinically useful, due to the cardiovascular protection elicited by the increased activity of angiotensin(1–7), thereby attenuating angiotensin II effects on vasoconstriction and sodium retention [31, 34]. *In vivo* ACE-2 deficiency results in augmented vascular inflammation and plaque instability [36], while its overexpression has been also shown to reduce left ventricular damage during myocardial infarction [37]. It has been additionally suggested that ACE-2 plays a key role in the reduction of left ventricular remodelling as well as in preserving ventricular function in humans [38].

**Fig. 2:**
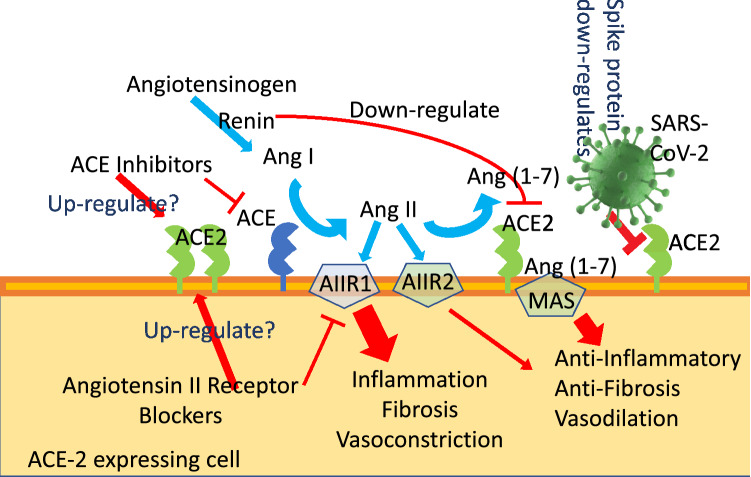
RAAS pathway and drugs that target ACE and ARB

Also sex seems to play a role in the pathogenesis of SARS-CoV-2 infection, with most of CoViD-19-associated deaths having been recorded among male individuals both in China (70–73%) [11, 39] and in Italy (64%) [8]. The fact that the ACE-2-encoding gene is located on chromosome X [40] could be a relevant factor in this regard. The SARS-CoV-2 uses the transmembrane protease serine 2 (TMPRSS2), an androgen-regulated gene, for viral spike protein priming [1]. ACE-2 receptor polymorphisms have, *in silico*, different affinities for the spike proteins SARS-CoV-2 [41–43]. Another confounding factor is smoke, with cigarette smoking increasing ACE-2 expression not only in experimental models [44] but also in humans [45] notwithstanding the well-established immunomodulatory effects of nicotine. Respiratory tract inflammation can expand ACE-2 receptor availability for respiratory cell infection by SARS-CoV-2. This point is also controversial [46], with the percentage of COVID-19-affected patients admitted to intensive care units in China being almost double in current smokers than in non-smokers [10].

These findings provide important insights into the molecular bases of the broad cross-talk between RAAS (Fig. 2) and SARS-CoV-2 cellular recognition and infection [1–3]. Experimental studies on Lewis rats have documented a 4.7-fold increased expression of cardiac ACE-2 gene after treatment with the ACE-inhibitor lisinopril, and by 2.8-fold after treatment with the angiotensin II (AT)1 receptor blocker losartan; however, ACE-2 activity remained paradoxically unchanged with lisinopril, being increased after losartan administration [47]. In spontaneously hypertensive rats treated with losartan, ACE-2 increased in renal but not in cardiac tissues [48]. In contrast, patients treated with ACE-Is and ARBs show an ACE-2 down-regulation at the mRNA and protein levels in kidney and cardiac tissues [49]. Angiotensin II is also able to up-regulate ACE and down-regulate ACE-2 in human kidney tubular cells, which were blocked by an AT1 receptor antagonist (losartan), but not by an AT2 receptor blockade [49].

These experimental observations have raised concerns that ongoing treatments with ACE-Is or ARBs could worsen the prognosis of COVID-19-affected patients suffering from simultaneously occurring cardiovascular disease conditions. In fact, it has been hypothesized that the ACE-2 overexpression induced by these two drug classes, although elicited by different mechanisms, could favour SARS-CoV-2 human lung tissue colonization [50]. By contrast, it has been additionally claimed that both ARBs [51] and ACE-Is [29] could result potentially useful in the clinical course of SARS-CoV-2-infected patients [34] (Fig. 2).

Due to the huge number of hypertensive, cardiovascular disease-affected and diabetic people currently treated with ACE-Is or ARBs, many of whom are now requesting a strong reassurance by their Cardiologists, we think it would be necessary to clarify the contents of such a relevant debate from a practical/clinical viewpoint.

The recent HFSA/ACC/AHA Statement [52] has claimed the need of continuation of ongoing treatments with RAAS antagonists in patients taking these drugs for various cardiovascular conditions, diabetic nephropathy or hypertension, despite theoretical concerns that their use might worsen the COVID-19 clinico-pathological evolution, should these subjects become infected by SARS-CoV-2. Also the Position Statement of the Council on Hypertension of the European Society of Cardiology [53] strongly suggests that, since SARS-CoV-2 binds to ACE-2 enzyme to infect host’s cells, and although ACE-2 levels are increased following treatment with ACE-Is and ARBs, there is currently lack of evidence supporting in humans the harmful effects of ACE-Is and ARBs in the context of the COVID-19 pandemic, thereby recommending that physicians and patients should continue ongoing treatments with their usual anti-hypertensive therapies.

Among the COVID-19-affected, deceased Italian patients, 24% of those followed were under treatment with ACE-Is and 16% with ARBs [8], with almost 70% of them also showing pre-existing hypertension. With the aim of reinforcing the above-reported statements, some very recent studies further contribute to clarify the debate [54–56]. In a retrospective study on 1128 hospitalized COVID-19-affected patients with hypertension, including patients taking ACE-Is (17%) or ARBs (83%) with a median age of 64–66 years (about 52% of whom males) and an additional 940 COVID-19-affected patients treated by other anti-hypertensive drugs with a median age of 64 years (about 53% of whom males), the unadjusted case-fatality rate was either significantly lower in the former group versus the non-ACE-Is/ARB group (3.7% vs. 9.8%, *P* = 0.01) [55, 56], or both groups were equal in terms of severe disease and deaths [54]. Nevertheless, the Chinese cohorts consisted of younger patients and had less comorbidities than the Italian CoViD-19-affected patients who succumbed to the disease. Interestingly, the platelet count was significantly lower [55], similarly to the prothrombin time [54] and the D-dimer [56] levels, in the ACE-Is- or ARB-treated, COVID-19-affected patients. After multivariate analysis for age, sex, comorbidities, and in-hospital medications [56], the adjusted HR for the ACE-Is/ARB group was 0.42 (95% CI 0.19–0.92; *P* = 0.03). The cautious conclusion of the authors, due to the intrinsic limitations of a retrospective study, was that “it is unlikely that in-hospital use of ACE-inhibitors/ARBs was associated with an increased mortality risk” [56]. Four very recent retrospective studies did not find any influence of the two RAAS-inhibitors on the clinical course [57], the rate of in-hospital death [58] or propensity of developing COVID-19 infection [59, 60].

As a conclusive remark, we can summarize “the current state-of-the-art” by underscoring and expressing endorsement on the statements independently expressed by HFSA/ACC/AHA and the Council on Hypertension of the European Society of Cardiology with just one sentence: “*Further data are needed in order to draw definitive conclusions*”.

Conclusions: Due to the large number of older patients treated all over the world with RAAS-antagonists for hypertension, diabetic nephropathy and severe cardiovascular disease conditions, although both authoritative statements and some preliminary contributions seem to dispel the fear that RAAS-antagonists could worsen the clinical course of COVID-19-affected patients, we claim the need for adequately powered, prospective studies aimed at providing an “ad hoc” answer to the following relevant questions:

1. Do ACE-Is and ARBs display similar or quantitatively divergent effects in COVID-19 patients?
2. Could ACE-Is or ARBs eventually be resulting in neutral, useful, or even dangerous in terms of cardiovascular protection in the clinical course of SARS-CoV-2 frail, comorbid infected patients?
3. How they affect infection of the numerous different type of ACE2 positive cells in the organism throughout the human body?

At present, insufficient detailed data from trials have been made available.
